# Identifying the effect of patient sharing on between-hospital genetic differentiation of methicillin-resistant *Staphylococcus aureus*

**DOI:** 10.1186/s13073-016-0274-3

**Published:** 2016-02-13

**Authors:** Hsiao-Han Chang, Janina Dordel, Tjibbe Donker, Colin J. Worby, Edward J. Feil, William P. Hanage, Stephen D. Bentley, Susan S. Huang, Marc Lipsitch

**Affiliations:** Department of Epidemiology, Center for Communicable Disease Dynamics, Harvard T.H. Chan School of Public Health, Boston, MA USA; Pathogen Genomics, The Wellcome Trust Sanger Institute, Wellcome Trust Genome Campus, Hinxton, CB10 1SA UK; Department of Biology, Drexel University, Philadelphia, PA USA; Nuffield Department of Medicine, University of Oxford, Oxford, UK; Department of Biology and Biochemistry, University of Bath, Bath, BA2 7AY UK; Division of Infectious Diseases and Health Policy Research Institute, University of California Irvine School of Medicine, Irvine, CA USA

**Keywords:** Genetic differentiation, Patient sharing, Gene flow, Transmission, *Staphylococcus aureus*

## Abstract

**Background:**

Methicillin-resistant *Staphylococcus aureus* (MRSA) is one of the most common healthcare-associated pathogens. To examine the role of inter-hospital patient sharing on MRSA transmission, a previous study collected 2,214 samples from 30 hospitals in Orange County, California and showed by *spa* typing that genetic differentiation decreased significantly with increased patient sharing. In the current study, we focused on the 986 samples with *spa* type t008 from the same population.

**Methods:**

We used genome sequencing to determine the effect of patient sharing on genetic differentiation between hospitals. Genetic differentiation was measured by between-hospital genetic diversity, *F*_*ST*_, and the proportion of nearly identical isolates between hospitals.

**Results:**

Surprisingly, we found very similar genetic diversity within and between hospitals, and no significant association between patient sharing and genetic differentiation measured by *F*_*ST*_. However, in contrast to *F*_*ST*_, there was a significant association between patient sharing and the proportion of nearly identical isolates between hospitals. We propose that the proportion of nearly identical isolates is more powerful at determining transmission dynamics than traditional estimators of genetic differentiation (*F*_*ST*_) when gene flow between populations is high, since it is more responsive to recent transmission events. Our hypothesis was supported by the results from coalescent simulations.

**Conclusions:**

Our results suggested that there was a high level of gene flow between hospitals facilitated by patient sharing, and that the proportion of nearly identical isolates is more sensitive to population structure than *F*_*ST*_ when gene flow is high.

**Electronic supplementary material:**

The online version of this article (doi:10.1186/s13073-016-0274-3) contains supplementary material, which is available to authorized users.

## Background

Methicillin-resistant *Staphylococcus aureus* (MRSA) is a leading cause of hospital-associated infections [1–4], with around 75,000 invasive MRSA infections reported in the United States in 2012 [5]. MRSA colonizes sites including the axilla, groin, gastrointestinal tract, and nares, and is typically spread via skin-to-skin contact, or contaminated medical devices [6, 7]. In hospitalized patients, MRSA causes a wide breadth of infections, including skin and soft-tissue infections, pneumonia, endocarditis, septic arthritis, osteomyelitis, device-associated infections, bacteremia, and sepsis [8]. Risk factors for MRSA infections include previous hospitalization, wounds, invasive medical devices, and immune system impairment [9, 10].

Understanding transmission dynamics within and between hospitals, between community and hospital, and within the community is important for disease control. Transmission-dynamic modeling has suggested that an MRSA outbreak in one facility contributes to MRSA prevalence in other connected healthcare facilities [11–14]. Different scales of genetic data have been used to study within- and/or between- hospital transmission. Ke *et al.* collected samples from 30 hospitals in Orange County, California and showed by *spa* typing that genetic differentiation decreases significantly with patient transfer between hospitals [15]. Using genome sequencing data, Long *et al.* found no evidence of within-hospital transmission between patients with sterile-site infections in four hospitals in Houston [16] and Prosperi *et al.* reported no phylogeographic clustering of samples from the same hospitals in northeast Florida [17].

The *spa* typing method involves the sequencing of a polymorphic variable-number tandem repeat within the 3′ coding region of the protein A-encoding gene (*spa*) and is one of the standard tools for MRSA surveillance studies [18–21]. Protein A binds immunoglobulins, and due to its important function in host-parasite interaction, demographic effects inferred from *spa* typing can possibly be biased by natural selection. More importantly, it has been suggested that the limited variation in *spa* typing hampers its power to detect spatial spread over local scales [22–24]. Although Ke *et al.* [15] successfully identified the effect of patient sharing in a local setting (Orange County, California) using *spa* typing, with most samples having the same *spa* type t008, the signal relied on the unusual *spa* types and might not reflect the overall transmission dynamics. Here, we focused on *spa* type t008/USA300, the dominant community associated clone in the United States [25–27], and used higher-resolution genome-sequencing data of isolates from the same hospitals as [15, 28] to examine transmission dynamics and the association between genetic differentiation and patient sharing. We compared the power of different tools that characterize genetic differentiation when applied to genome sequencing data of the MRSA population on the county level. We also investigated the factors associated with within- and between-hospital genetic diversity. Our goal was both to assess whether the results of Ke *et al.* were replicated using genomic data, and to compare measures of population substructure for their ability to detect migration of bacteria – in this case assumed to be via patient transfer from the community and between hospitals – using different kinds of genetic/genomic data.

## Materials and methods

### Sample selection

A total of 986 methicillin-resistant *Staphylococcus aureus* isolates assigned as USA300 collected between 2008 and 2010 from 30 hospitals in Orange County, California, USA were selected from a previously published study [15, 28]. Hospitals were instructed to provide isolates from unique patients. The sample sizes and the numbers of hospital- and community-onset isolates are shown in Additional file 1: Table S1. An isolate was considered to be hospital-onset if the difference between admission date and the culture date was greater than 2 days. Community-onset in this study includes both true community-onset infections and infections in post-discharge facilities (healthcare-associated community onset (HA-CO)) because we were not able to distinguish them.

### Genome sequencing, SNP calling, and phylogenetic reconstruction

DNA was extracted using the QIAamp DNA Mini Kit (Qiagen) and core genomes were sequenced using Illumina HiSeq2000 with 100 bp paired-end reads. Reads were mapped against the USA300 reference sequence FPR3757 (accession NC_007793) using SMALT v0.5.8 (http://www.sanger.ac.uk/science/tools/smalt-0) with subsequent realignment around indels using GATKv1.5.9 [29]. The average depth of reads is 115. Single nucleotide polymorphisms (SNPs) were called using samtools and subsequently filtered to remove sites with a quality score less than 50, less than four reads covering the SNP site, and a SNP/mapping quality ratio less than 0.75. SNPs in repeat regions identified using RepeatScout [30] and mobile genetic elements were excluded. This resulted in 24,660 SNPs from the core genome. Sequence data were deposited in the European Nucleotide Archive (project accession PRJEB2686; for isolate accessions see Additional file 2: Table S2).

Maximum likelihood as implemented in RAxML v0.7.4 [31] with the GTRGAMMA model and 100 bootstrap replications was used to reconstruct a phylogenetic tree of HA-onset isolates. The tree was plotted using iTOL v3.0 [32] and branches and tips were colored according to the hospital where isolates were collected.

### Patient sharing between hospitals

As in Ke *et al.* [15], patient sharing from hospital A to hospital B was calculated by

$$ {P}_{A->B}\kern0.5em =\kern0.5em {m}_{A->B}/{N}_B $$

where *N*_*i*_ represents the number of admissions in hospital *i* per year and *m*_*i->j*_ is the number of patients transferred from hospital *i* to hospital *j* per year. We calculated the number of patients transferred from hospital *i* to hospital *j* by summing the numbers of direct and indirect patient transfers. Patient sharing between any two hospitals A and B was calculated by the taking the average between two directions:

$$ {M}_{AB}\kern0.5em =\kern0.5em \frac{P_{A\to B}\kern0.5em +\kern0.5em {P}_{B\to A}}{2}. $$

### Genetic differentiation

We used three statistics to characterize genetic differentiation between hospitals: average pairwise difference (*π*) between isolates from different hospitals, *F*_*ST*_, and the proportion of nearly identical isolates (*I*). *F*_*ST*_ is based on the variance of allele frequencies between populations [33] and was calculated using the R package *Hierfstat* [34]*.* The sample sizes for each hospital range from 1 to 68. Hospitals with sample sizes smaller than 10 were excluded in the analysis of *F*_*ST*_.

The proportion of nearly identical isolates between hospitals (*I*) is determined by the proportion of isolate pairs with smaller than 0.15 % differences among all the SNPs (equivalent to fewer than 37 SNP differences) between hospitals. This threshold is similar to the 40-SNP threshold used to discount direct transmission in previous studies [16, 35, 36]. Given that the mutation rate is 1.22 × 10^-6^ per site per year for USA300 [37] and the size of core genome is 2.5 Mb, the divergence per year is about three SNPs. Thirty-seven SNPs divergence between two genomes therefore corresponds to approximately 6.16 (=37/2/3) years on two lines of descent from the most recent common ancestor, indicating that the maximum divergence time for isolates we are counting as ‘nearly identical’ is about 6 years for the threshold of 37 SNPs and about 4 years for the lower threshold of 25 SNPs considered in sensitivity analyses. These divergence times are upper bounds given that (1) we consider SNP distances up to the threshold as ‘nearly identical’ and (2) short-term mutation accumulation of bacteria occurs faster than long-term evolutionary rates, due to the survival of weakly deleterious mutations over short but not long time scales [38].

Within-hospital genetic diversity was calculated by averaging the proportion of SNP differences between all pairs of isolates from the same hospital and singleton SNPs were excluded to minimize the effect of potential sequencing error and sample size.

### Permutation tests

To assess statistical significance of observed correlations, test statistics were recalculated for 10,000 random permutations of the data, in each of which the hospital identifier list was permuted relative to the list of isolates.

### Coalescent simulation

Coalescent simulation was performed using program *ms* [39]. We assume no recombination, constant population size, an infinite-sites model (all polymorphic sites are biallelic) and no within-host evolution. We used the ‘steady-state’ number of patients (N^*^) as population size in each hospital. N^*^ was calculated by the number of admissions in each hospital in 1 year times the average length of stay divided by 365 days. In addition, we assumed that there was a subpopulation with population size N^*^ = 5000, representing the community, and its sample size was 0. The sample sizes used in coalescent simulations were the same as the sample sizes in the data. We assumed that the mutation rate is eight per genome per year [40] and that the generation time is equal to the average of length of stay = 9 days.

We simulated four scenarios: (1) high patient sharing and high community contribution; (2) high patient sharing and low community contribution; (3) low patient sharing and high community contribution; and (4) low patient sharing and low community contribution. For high patient sharing (1 and 2), empirical patient sharing from Orange County was used for migration rates between subpopulations in the coalescent model; for low patient sharing (3 and 4), migration rate was equal to empirical patient sharing from Orange County divided by 100. The number of replicates for each model was 100. The proportion of patients in each hospital that are from the community (*C*_*from*_), and the proportion of infections in the community that are from each hospital (*C*_*to*_) are listed in Table 1.

**Table 1:** Parameter values for coalescent simulations

Model	Migration rate between hospitals	Community contribution	
		*C*_*from*_^a^	*C*_*to*_
1	Empirical patient sharing between hospitals	50 %	3 %
2	Empirical patient sharing between hospitals	5 %	1 %
3	One-100th of empirical patient sharing between hospitals	0.5 %	0.03 %
4	One-100th of empirical patient sharing between hospitals	0.05 %	0.01 %

^**a**^ *C*_*from*_ is the proportion of patients in each hospital that are from the community, and *C*_*to*_ is the proportion of infections in the community that are from each hospital

In addition to infinite-sites model, we also performed coalescent simulations for a single microsatellite marker using the infinite-allele model and a stepwise mutation model [41] in order to compare a single site-multiple alleles microsatellite marker with multiple site-biallelic SNPs. The mutation rate of microsatellites is known to be higher than that of point mutations [42], and therefore we used 10^4^- and 10^6^-times the per-site point mutation rate as the mutation rate for microsatellite model.

## Results

### Within-hospital and between-hospital genetic diversity

A total of 986 MRSA isolates were sequenced from 30 hospitals in Orange County in 2008 to 2010, across which 24,660 polymorphic sites were identified in the core genome.

The average pairwise genetic distance between samples from the same hospitals was significantly smaller than that between samples from different hospitals (0.353 % vs. 0.357 % of all SNP positions, or 87 and 88 SNP differences; permutation test (*n* = 10,000), *P* value = 0.0045; Additional file 1: Figure S1A), though the difference between them was small. SNP differences in this range indicate that the isolates are about 15 years (=87/2/3 and 88/2/3) divergence between each other. Among all the isolate pairs with no SNP differences, 66 % (31 out of 47) of them were from the same hospital. Among these 31 pairs from the same hospital, 17 pairs of isolates involve hospital-onset isolates (at least one was isolated after day 2 of the hospital stay), suggesting transmission, and 10 out of 17 pairs of isolates were collected in the same month (Additional file 1: Figure S2). Although the nearest neighbors of some isolates in the phylogeny are from the same hospital, the phylogeny of all hospital-onset isolates shows no visual evidence of clustering between isolates from the same hospitals (Additional file 1: Figure S3). Together, the distributions of within and between hospital pairwise distance (Additional file 1^1^ Figure S1A) and the phylogeny (Additional file 1: Figure S3) suggest that gene flow between hospitals facilitated by patient sharing between hospitals diluted the genetic structure to the point that pairwise genetic diversity cannot be used to distinguish isolates from the same or different hospitals.

### Predictors of within-hospital genetic diversity

We tested the factors that were associated with within-hospital genetic diversity. Because estimates of the within-hospital genetic diversity are sensitive to the sample size (Pearson’s correlation test between within-hospital genetic diversity and sample size, *r* = 0.376, *P* value = 0.045), we calculated the partial correlation between within-hospital genetic diversity and other factors when controlling for the sample size and excluded four hospitals with a sample size of less than five from analysis.

The number of admissions per year (ranging from 1,068 to 30,930) and the proportion of community-onset isolates (ranging from 56 % to 100 %) were not significantly correlated with within-hospital genetic diversity (*P* values = 0.41 and 0.10). The number of hospitals that a hospital receives patients from (indegree) and the proportion of patients from other hospitals were both positively correlated with within-hospital genetic diversity (Pearson partial correlation coefficients = 0.587 and 0.563, *P* values = 0.00051 and 0.0011, respectively) (Additional file 1: Figure S4). The indegree and the proportion of patients from other hospitals were significantly positively correlated with each other (Pearson’s correlation *r* = 0.562, *P* value = 0.0028).

### Patient sharing as a predictor of genetic differentiation between pairs of hospitals

We used three methods to characterize genetic differentiation between hospitals: average pairwise difference (*π*) between isolates from different hospitals, the fixation index *F*_*ST*_, and the proportion of nearly identical isolates (*I*), which is defined as the proportion of isolate pairs with smaller than 0.15 % differences (equivalent to smaller than 37 SNPs) among all the SNPs between a pair of hospitals. A similar threshold, 40 SNPs, was used to discount direct transmission between individual patients in previous studies [16, 35, 36].

First, we compared genetic differentiation between hospitals with and without patient sharing. The proportion of nearly identical isolates between hospitals with patient sharing was significantly larger than that between hospitals without patient sharing (median = 0.0055 vs. 0; permutation test (*n* = 10,000), *P* value = 0.008, Additional file 1: Figure S5). *F*_*ST*_ and the average pairwise difference *π* between hospitals with patient sharing were not significantly smaller than those without patient sharing (permutation test (*n* = 10,000), *P* values = 0.136 (*F*_*ST*_) and 0.900 (*π*)).

**Fig. 1:**
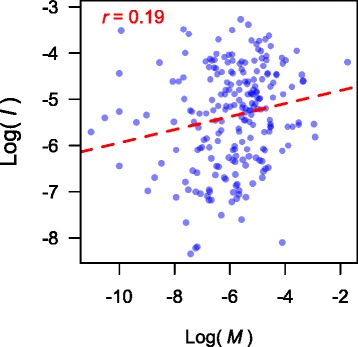
The proportion of nearly identical isolates increases with the level of patient sharing (Pearson’s correlation *r* between log(*M*) and log(*I*) = 0.185, Mantel test *P* value = 0.038; *I* and *M* are the proportion of nearly identical isolates and the level of patient sharing, respectively)

Next we estimated the association between genetic differentiation and the level of patient sharing (*M*). The proportion of nearly identical isolates between hospitals was significantly positively correlated with the level of patient sharing (Pearson’s correlation *r* between log(*I*) and log(*M*) = 0.185, Mantel test *P* value = 0.038; Fig. 1). The results were relatively insensitive to the choice of SNP difference cutoff values used to define nearly identical isolates (Additional file 1: Figure S6). The correlation between *F*_*ST*_ and the level of patient sharing was weaker and not statistically significant (Pearson’s correlation *r* of log(*M*) and log(*F*_*ST*_) = -0.112, Mantel test *P* value = 0.11), and the same applied to the correlation between the average pairwise difference and the level of patient sharing (Pearson’s correlation *r* of log(*M*) and *π* = 0.085, Mantel test *P* value = 0.20).

### Examining discrepancies between results with different measures of genetic differentiation

**Fig. 2:**
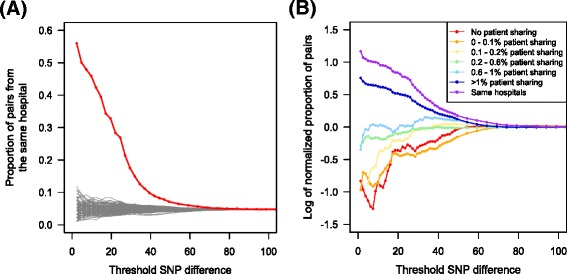
Isolate pairs with smaller SNP differences were more likely to come from the same hospital or hospitals with higher level of patient sharing. **a** Isolate pairs with smaller SNP differences were more likely to come from the same hospital (red line) than 100 permutations of random assignment of hospitals (gray lines). **b** In order to obtain the effect of different levels patient sharing, we calculated normalized proportion of pairs, which is the quantity (*N*_*ki*_/*N*_*i*_)/(*N*_*k*_/*N*), where *N* is the total number of pairs of isolates, *N*_*k*_ is the number of pairs of isolates from hospitals with a particular amount of patient sharing *k*, *N*_*i*_ is the number of pairs of samples with less than *i* SNP differences, and *N*_*ki*_ is the number of pairs of samples coming from hospitals with a particular amount of patient sharing *k* differing by less than *i* SNPs. Samples collected from the hospitals with higher level of patient sharing were more likely to have smaller SNP difference. Even a very low level of patient sharing (0.1-0.2 %) shows higher normalized proportion of pairs with smaller SNP differences than no patient sharing

Isolate pairs with smaller SNP differences were more likely to come from the same hospitals or hospitals with a higher level of patient sharing (Fig. 2), suggesting that patient sharing transmits strains between hospitals. We hypothesized that the lack of significant association between patient sharing and *F*_*ST*_ or *π* is because these measures are less powerful than the proportion of nearly identical isolates for detecting population structure when gene flow between populations is high, as in the case here, since the latter is particularly sensitive to detecting recent transmission events. For example, in Wright’s island model with the same subpopulation sizes and migration rates among them [43], *F*_*ST*_ at equilibrium is approximately 1/(1 + 2 *Nm*), where *N* is the size of each subpopulation and *m* is the migration rate between subpopulations [44]. It is therefore expected that when *Nm* is large, *F*_*ST*_ is not very sensitive to each unit change in *Nm*. When patient sharing is high, exchange of alleles between hospitals is expected to be frequent, and allele frequencies in different hospitals tend to be similar. In this case, the impact of genetic drift and sampling error on allele frequencies can be similar to that of patient sharing. Because *π* and *F*_*ST*_ are based on allele frequencies, their powers to detect the effect of patient sharing is lower.

**Fig. 3:**
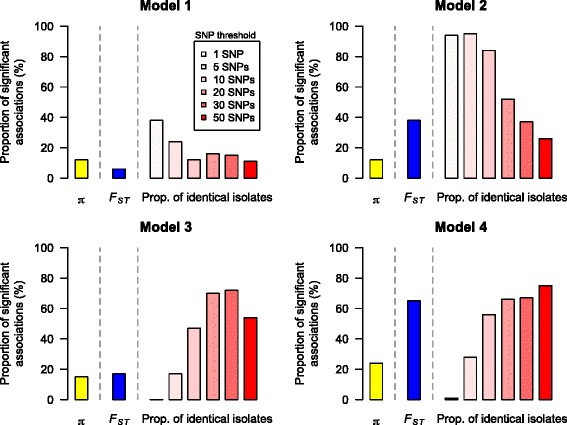
The power of *π*, *F*_*ST*_, and the proportion of nearly identical isolates to detect the effect of patient sharing. The proportion of nearly identical isolates is more powerful than *π* and *F*_*ST*_ if the threshold for nearly identical isolates is chosen properly. *F*_*ST*_ is more sensitive to changes in patient sharing if patient sharing is high (Model 4). *π* is less powerful in all four models here

We performed coalescent simulations to test our hypothesis. We simulated four scenarios: (1) high patient sharing (corresponding to migration between populations in the coalescent model) and high community contribution (corresponding to migration from an unsampled population with large population size); (2) high patient sharing and low community contribution; (3) low patient sharing and high community contribution; and (4) low patient sharing and low community contribution. The parameter values are described in Methods and shown in Table 1. The results show that when patient sharing between hospitals is high, either due to high patient transfer between hospitals (Model 2) or high level of community-onset infections in hospitals (Model 3) or both (Model 1), using the proportion of nearly identical isolates is more powerful than *F*_*ST*_ because it is sensitive to recent transmission events if proper SNP difference cutoff values are used (Fig. 3). If patient sharing is low (Model 4), the SNP difference between isolates from different hospitals is high and the proportion of nearly identical isolates is often 0 and less useful when the threshold is small (Fig. 3). The average pairwise difference is generally less powerful because it highly depends on allele frequency. For example, if allele frequencies in two hospitals are both 0.5, it suggests that genetic differentiation is low, but the average pairwise difference between hospitals in this case appears to be high (*π* =0.5). We also showed that the stochastic variation of *F*_*ST*_ and *π* between simulation runs is higher than that of the proportion of nearly identical isolates (Additional file 1: Figure S7).

### *F*_*ST*_ of microsatellite markers

Because we were extending an analysis to genomic data that had previously been performed with *spa* typing, we sought to understand how allele-frequency based analyses with single-locus markers compared to genome-wide, SNP-based analyses. The mutational process of the *spa* gene is complex, including deletion, duplication, and point mutations. For simplicity and generality, we compared the power of *F*_*ST*_ derived from a single-locus multiple-alleles microsatellite marker with that of multiple-locus biallelic SNPs to detect the effect of patient sharing. We ran computer simulations using two models for the microsatellite locus: an infinite alleles model and a stepwise mutation model. In the infinite alleles model, each mutation leads to a new allele; in stepwise mutation model, each mutation can either increase or decrease the number of sequence repeats by 1. We assumed the mutation rate in the microsatellite model is 10^4^ or 10^6^-fold higher than the per-site mutation rate in the multiple-locus SNP model.

When patient sharing is high and the contribution of strains from community-onset infections is relatively low (Model 2), *F*_*ST*_ calculated from microsatellite markers is more sensitive than *F*_*ST*_ calculated from multiple-locus biallelic SNPs (Additional file 1: Figure S8). When the community contribution is high (Models 1 and 3) the proportion of significant associations using *F*_*ST*_ calculated from microsatellite markers and using *F*_*ST*_ calculated from multiple-locus biallelic SNPs are similar and both small. When patient sharing and community contribution are both low (Model 4), multiple-locus biallelic SNPs perform better than microsatellite markers. The stochastic variation in *F*_*ST*_ of microsatellite markers is smaller than that of SNPs, and is smaller when mutation rate is higher (Additional file 1: Figure S7 and S9).

### Genetic differentiation and community-onset infections

If the hospitals are closer to each other, they are more likely to have overlapping community catchment areas. We hence hypothesized that community-onset infections in hospitals closer to each other would be similar genetically. The proportion of nearly identical isolates decreases with geographic distance (*D*) (Pearson’s correlation *r* between log(*I*) and *D* = -0.193, Mantel test *P* value = 0.086) and *F*_*ST*_ increases with geographic distance (Pearson’s correlation *r* between *F*_*ST*_ and *D* = 0.187, Mantel test *P* value = 0.076), though only borderline significant, suggesting that genetic differentiation increases with geographic distance. However, it is difficult to distinguish the effects of geographic distance and patient sharing on genetic differentiation, because geographic distance and patient sharing were highly correlated with each other (Pearson’s correlation *r* = -0.454, Mantel test *P* value = 0.0002). Ideally, we could separate hospital-onset (HO) and community-onset (CO) samples into two groups and test whether the association between genetic differentiation and geographic distance is higher in the CO group and the association between genetic differentiation and patient sharing is stronger in the HO group, but our sample sizes are not sufficient for performing these tests.

Moreover, we tested the effect of average CO proportions on genetic differentiation between hospitals. The correlation between the average CO proportion and *F*_*ST*_ (Pearson’s correlation *r* = -0.143, Mantel test *P* value = 0.20) and the correlation between the average CO proportion and the log of the proportion of nearly identical isolates (Pearson’s correlation *r* = 0.156, Mantel test *P* value = 0.21) were not significant. Because the effect of average CO proportions on genetic differentiation may depend on the level of overlapping communities, we calculated the partial correlation between average CO proportions and genetic differentiation given geographic distance between hospitals. The partial correlation of average CO proportion and genetic differentiation were still not significant after controlling for geographic distances between hospitals (log(*I*), Pearson’s correlation *r* = 0.174, Mantel test *P* value = 0.19; *F*_*ST*_, Pearson’s correlation *r* = -0.160, Mantel test *P* value = 0.16). The lack of statistically significant impact of CO proportion here could be due to the limited variation in CO proportion across hospitals (Additional file 1: Table S1).

## Discussion

In this study, we used genome sequencing data of 986 MRSA regional isolates to study MRSA transmission within and between hospitals and between hospitals and their surrounding community. We confirmed the impact of patient sharing on population structure [15] by showing a positive correlation between the proportion of nearly identical isolates between hospitals and the level of patient sharing. We found that many sample pairs without any SNP difference were from unique patients from the same hospital and their time of sample collection was very close, supporting the presence of within-hospital transmission, consistent with earlier findings that patient-to-patient transmission occurs, even if attentive infection prevention strategies are used [36].

### Identifying the effect of patient sharing

Although we detected a significant association between the proportion of nearly identical isolates and patient sharing, the association between *F*_*ST*_ and patient sharing was not significant. We propose that these different results might be due to a lack of power of *F*_*ST*_ when patient sharing and the contribution of community-onset infections are high, and we confirmed our hypothesis by performing coalescent simulations using parameters informed by empirical data. The association between patient sharing and *F*_*ST*_ calculated from *spa* types in Ke *et al.* [15] was likely attributed to the rare and more divergent isolates with *spa* types that were excluded from the present study. Although the variation in *spa* types is usually too low for detailed tracking of spatial spread in short-term local settings, if there is enough variation, it can potentially be powerful because when the rare or more divergent isolates were shared between hospitals, it was very likely due to patient sharing.

Only a certain amount of divergence can occur before a *spa* change causes the sample to be discarded from the t008-lineage dataset. If within-hospital diversity reaches the maximum expected saturation point for within-*spa* type diversity, *F*_*ST*_ is not a suitable measure for genetic differentiation between hospitals. Engelthaler *et al.* showed that within-*spa* type diversity can be in the order of thousands of SNPs [45], which is much greater than the maximum SNP difference (269 bp) in our dataset. This suggests that it is unlikely that the saturation of within-t008 diversity lowered the power of *F*_*ST*_ in our study.

It has been suggested that the cloud of diversity is a major issue in identifying person-to-person transmission links [46, 47]. We sequenced a single isolate from each patient and do not have the information of within-host genetic diversity. However, we are concerned about hospital-level rather than patient-level dynamics in this study, and because the importance of patient-to-patient transmission effects diminishes considerably at the group level [47], there is less concern about within-host diversity here. To directly explore the impact of within-host diversity, multiple within-host pathogen genomic sequences from a range of scenarios, together with comprehensive epidemiological data, would be required.

### Low level of recombination

*S. aureus* has been shown to be primarily clonal with relatively low levels of recombination [37, 48–50]. We used Gubbins [51] to detect recombination in our dataset, and identified six regions of recombination, which in average account for 0.00064 % of genome and 5.93 % of SNPs. We excluded these regions and repeated our within-hospital analysis of within-hospital genetic diversity and the association between the proportion of nearly identical isolates, *F*_*ST*_ and *π* with patient sharing, and the results are consistent with the results before removing recombination (Additional file 1: Table S3). Genealogy-based methods generally perform better than *F*_*ST*_ if there is no recombination [52], however, genealogy-based parametric methods, such as *BEAST* [53] or *MIGRATE-N* [54, 55], cannot be used for estimating migration rate between hospitals because the number of parameters is too high (870 if using non-symmetric migration rates and 435 if using symmetric migration rates). Moreover, many pairs of sister strains on the tips of the phylogeny comes from different hospitals (Additional file 1: Figure S3), suggesting that many branches would have multiple migration events. Therefore, even if parametric methods were used to reduce the number of separate migration rates to estimate, the inference of rates is less reliable and many combinations of estimates might fit the data equally well.

### Star-like phylogeny

The phylogenetic tree we constructed shows relatively long external branches compared with internal branches (Additional file 1: Figure S3). A similar shape of phylogeny has also been seen in other studies of *S. aureus* in the United States [37, 56]. There are five possible explanations for star-like phylogeny: recombination [57, 58]; sequencing error; population expansion [59]; selective sweep [60]; and long-term colonization. The phylogeny after removing recombination regions detected by Gubbins is still star-like (Additional file 1: Figure S10), suggesting that recombination is unlikely to be the reason. We could not entirely rule out the possibility of sequencing error, but because we were still able to find several pairs of identical isolates, we think it does not play a major role in our dataset. Given that USA300 is a recently emerging clone [25], it is possible that population expansion and/or a selective sweep leads to the longer external branches. To test this hypothesis and to explore possible mechanisms resulting in such dynamics, further research would be required. Finally, long-term persistence in the host can lead to long external branches in the phylogeny [61], and because MRSA colonization sometimes persist for a long time [62], intra-host evolution can potentially explain part of the pattern seen here.

### Comparing genome-wide SNP with a single microsatellite marker

Our simulation results also indicate that, when *F*_*ST*_ is used, genomic SNP data are not always more powerful than microsatellite markers (though the proportion of nearly identical isolates identified by genome-wide SNP data is more powerful than microsatellite *F*_*ST*_ in our four models). When there is no recombination, there is one single evolutionary tree for all loci, and *F*_*ST*_ calculated from genome-wide SNP does not benefit from taking the average of multiple partially independent trees as it would in organisms with frequent recombination. Microsatellite markers are more sensitive to recent events than to events in the distant past because each new mutation can potentially lead to a new allele and the number of mutations (or the divergence time) between alleles is not trackable. Also, in the long term, a series of mutations can lead to convergence that would be misinterpreted as identity by descent [24, 63]. When patient sharing is high and community contribution is relatively low, microsatellite markers perform better than SNPs. In contrast, when patient sharing is low, the power of microsatellite markers is lower. Regions such as microsatellites that mutate rapidly are difficult to assay using next-generation sequencing methods based on short reads, but technological advances have the potential to greatly increase the read length [64], and we can expect that this will make these regions and their variation accessible to genomic analyses.

## Conclusions

With advances in sequencing technologies, very large samples of pathogen genomes are becoming available and can be used for studying disease transmission. Pathogen samples can be collected across different geographic scales, such as on the country, city, or hospital levels. Here we showed that for samples from different hospitals in the same county, the proportion of nearly identical isolates was more useful for detecting the effect of patient sharing than the classical statistic *F*_*ST*_ when using genomic data, and that *F*_*ST*_ calculated from genome sequencing data is not always more powerful than *F*_*ST*_ calculated from microsatellite markers.

### Availability of supporting data

The datasets supporting the results of this article are available in the European Nucleotide archive repository under accession PRJEB2686.

## Additional files

Additional file 1: **Supplementary materials.** Description: PDF file containing Figures S1-S10, and Tables S1 and Table S3. (PDF 14325 kb)

Additional file 2: **Table S2.** Description: European Nucleotide Archive accession number of each isolate. (XLSX 106 kb)

## Abbreviations

CO: community-onset
HO: hospital-onset
MRSA: methicillin-resistant *Staphylococcus aureus*
SNPs: single nucleotide polymorphisms
