# Supplementary material for: Identifying the effect of patient sharing on between-hospital genetic differentiation of methicillin-resistant Staphylococcus aureus
Source: Genome Med. 2016 Feb 13;8:18. doi: 10.1186/s13073-016-0274-3 (PMC4752745; doi:10.1186/s13073-016-0274-3)
Supplement: Supplementary file 1 — Supplementary materials. Description: PDF file containing Figures S1-S10, and Tables S1 and Table S3. (PDF 14325 kb) [file 13073_2016_274_MOESM1_ESM.pdf]

Supplementary materials

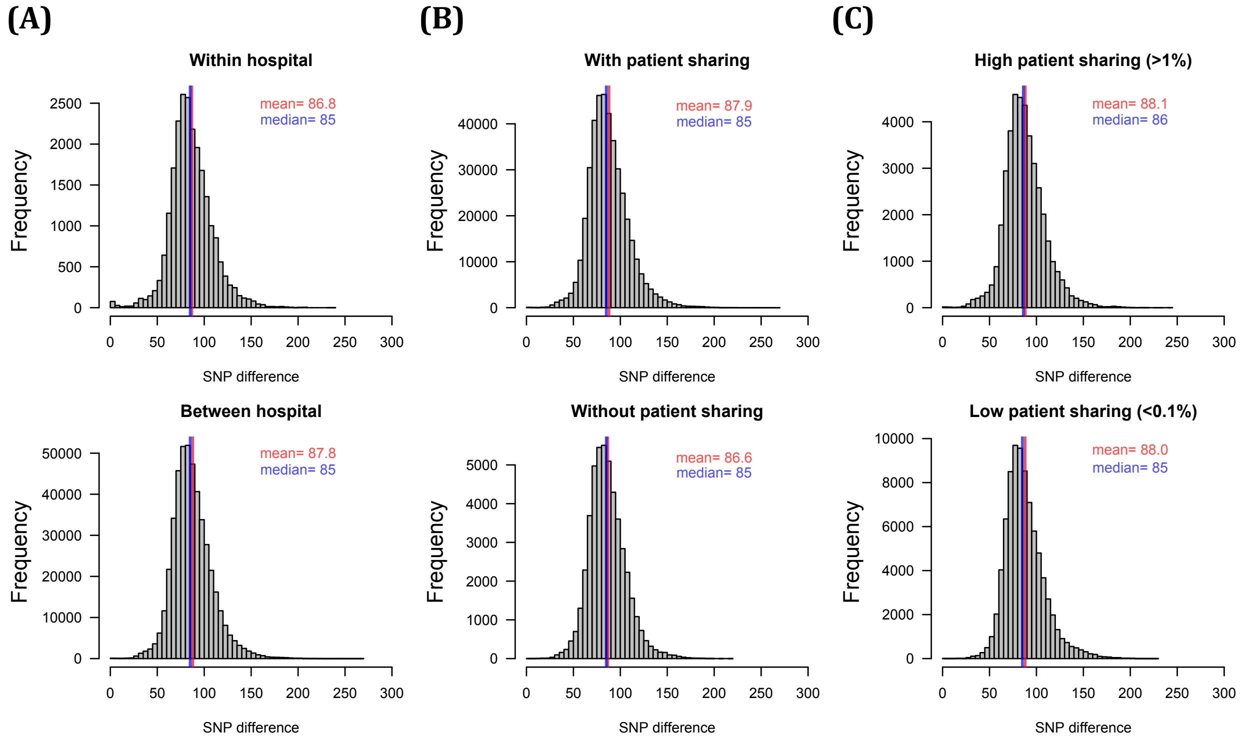

Figure S1. SNP differences between isolates. (A) SNP differences between isolates from the same or different hospitals. **(B)** SNP differences between isolates from the hospitals with and without patient sharing. **(C)** SNP differences between isolates from hospitals with high and low patient sharing.

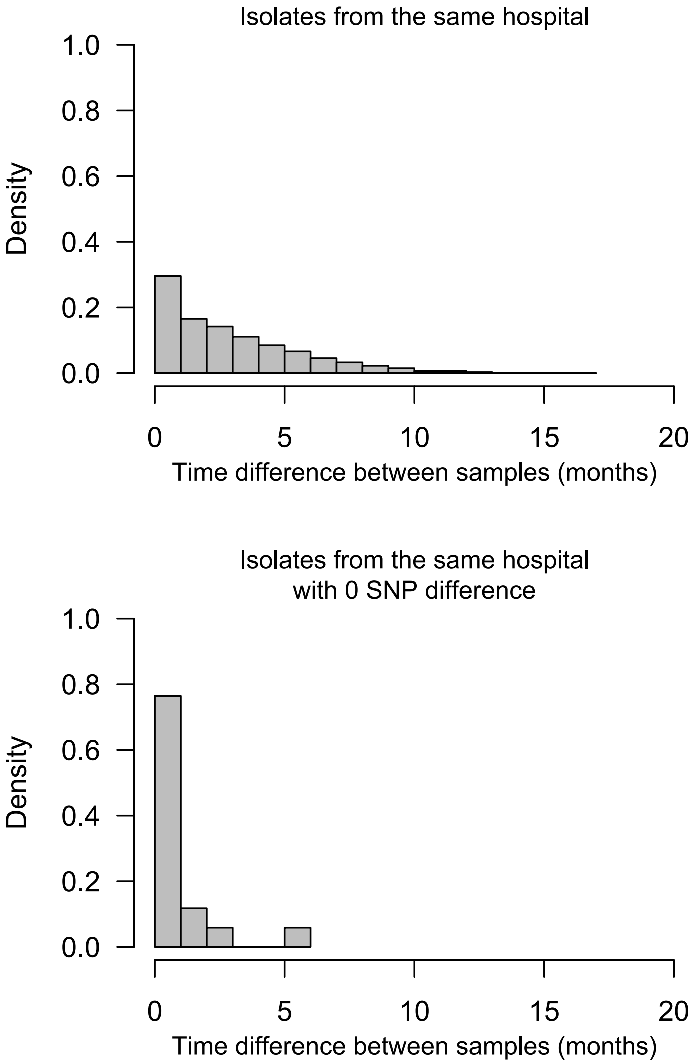

Figure S2. Time difference between samples. The difference in the time of collection between isolate pairs from the same hospitals with 0 SNP difference and at least one hospital-onset isolates (bottom) tends to be shorter than that of other isolate pairs.

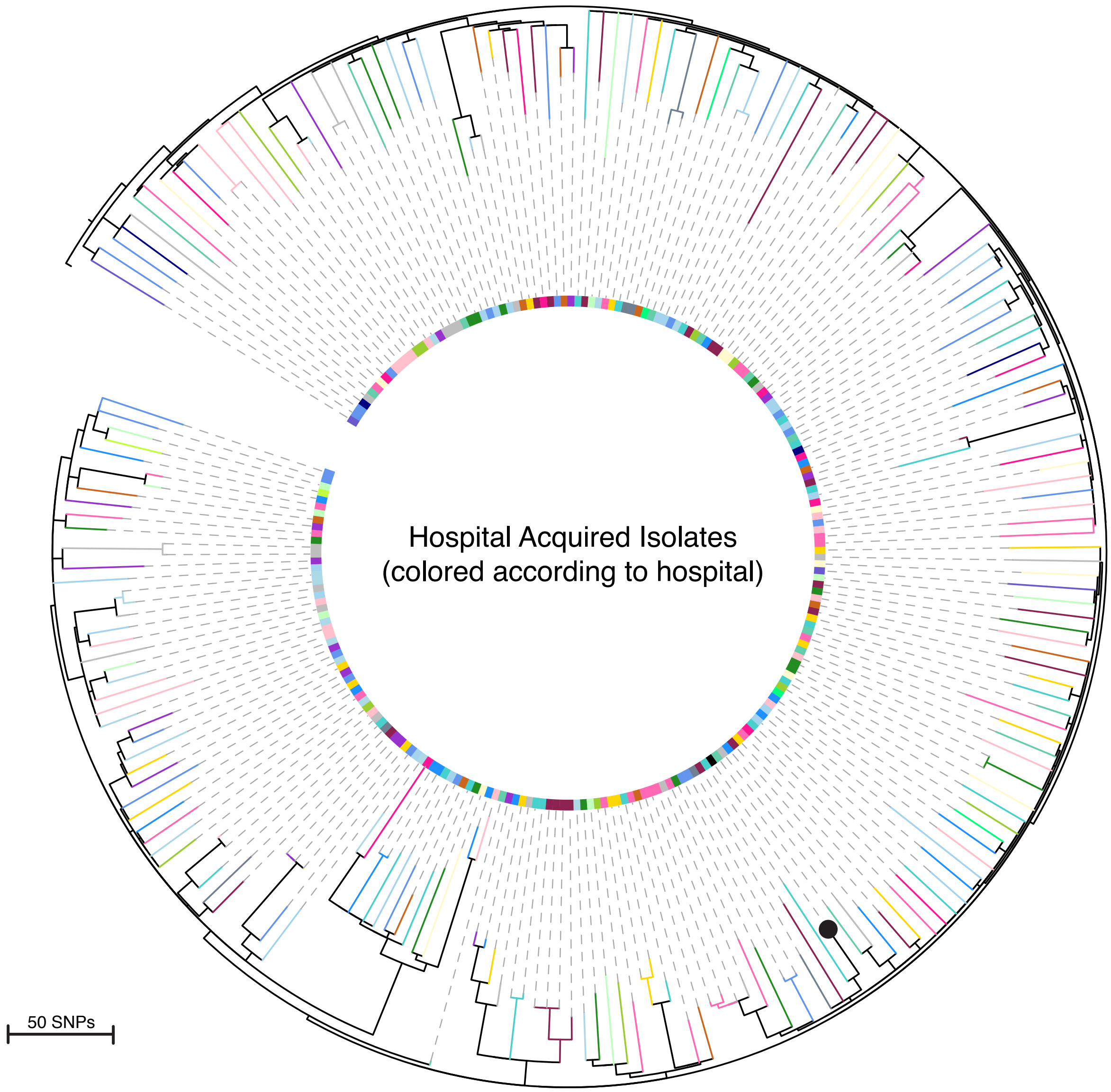

Figure S3. Rooted phylogenetic tree of hospital-onset isolates. Branch and inner ring colors represent the hospital origin of any given isolate. The black dot represents the position of the reference genome USA300 FPR 3757 (NC_007793). The tree was rooted using the distant isolate COL (CC8, ST250, accession NC_002951).

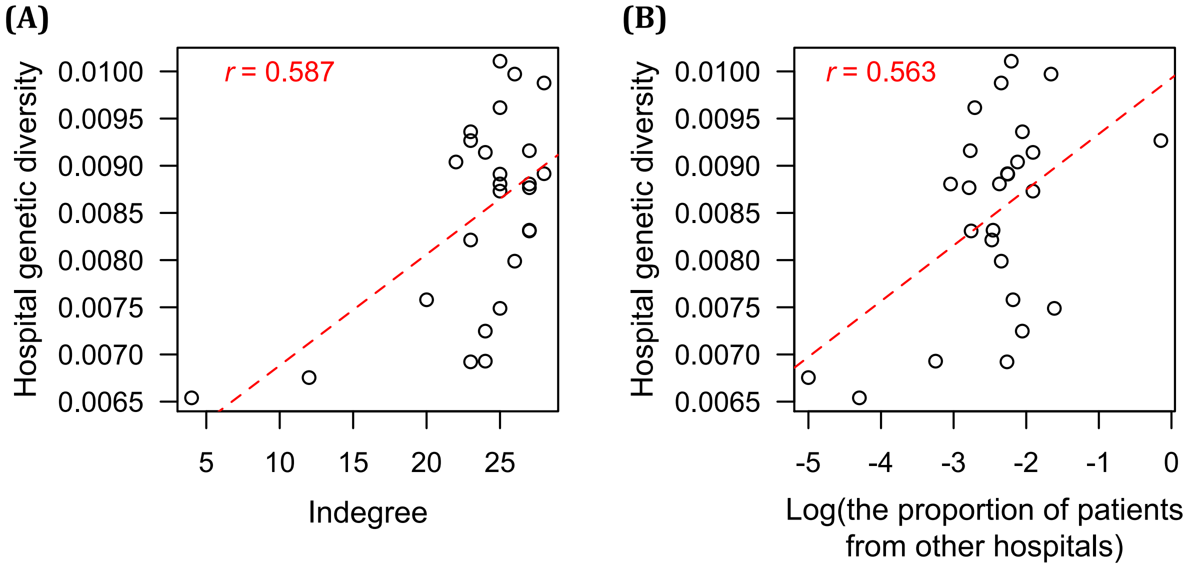

Figure S4. Within-hospital genetic diversity. Hospital genetic diversity was associated with **(A)** the number of hospitals a hospital receives patients from and **(B)** the proportion of patients from other hospitals. The correlation coefficient shown here is Pearson partial correlation coefficients when controlling for the sample size.

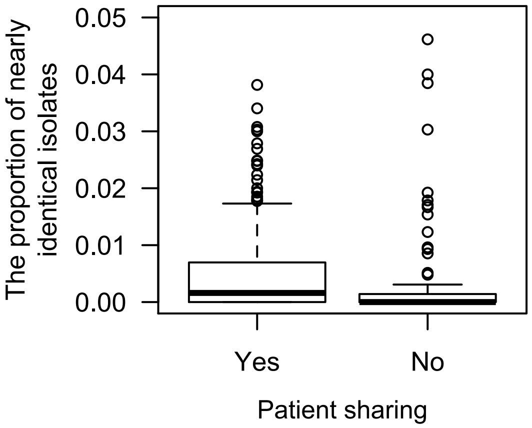

Figure S5. The proportion of nearly identical isolates between hospitals with patient sharing was significantly larger than that between hospitals without patient sharing (median= 0.0055 vs. 0; permutation test [*n* = 10000], *p*-value = 0.008).

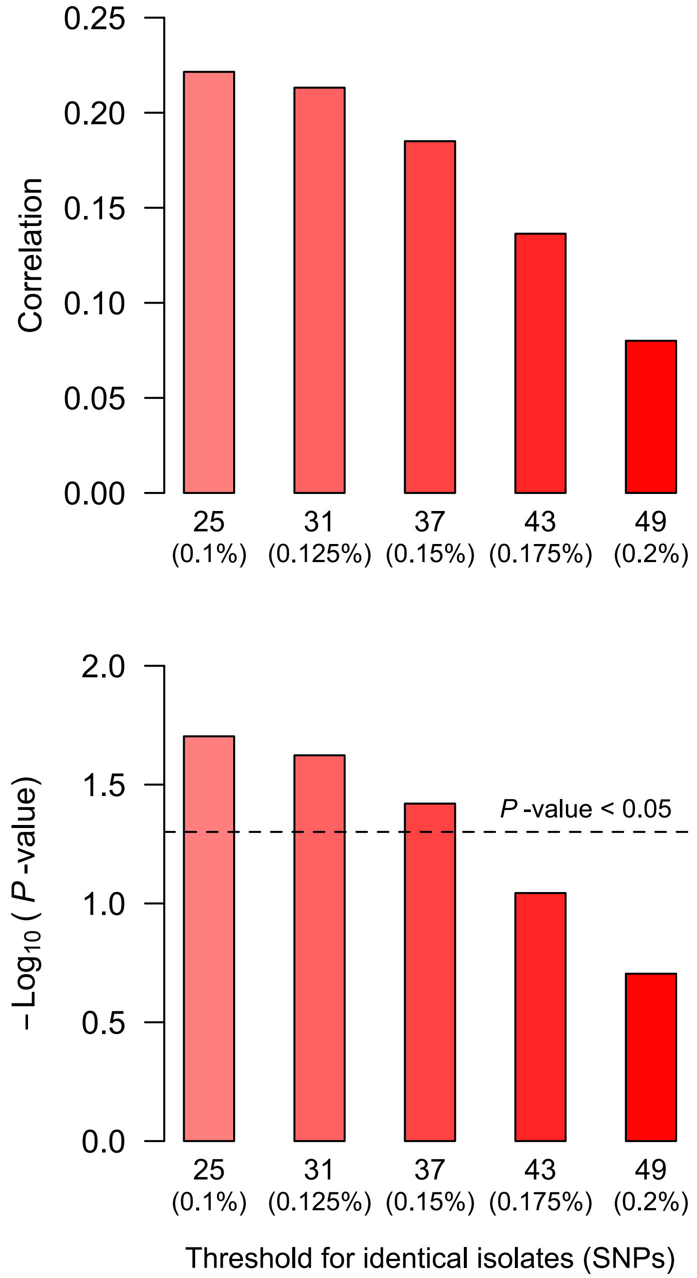

Figure S6. Correlation coefficients and *p*-values for different threshold values to define nearly identical isolates. The standard threshold used in this paper is 0.15% (37 SNPs). The results from SNP difference cutoff values different from 37 are similar. Color code corresponds to the different simulated cutoffs in Fig. 3.

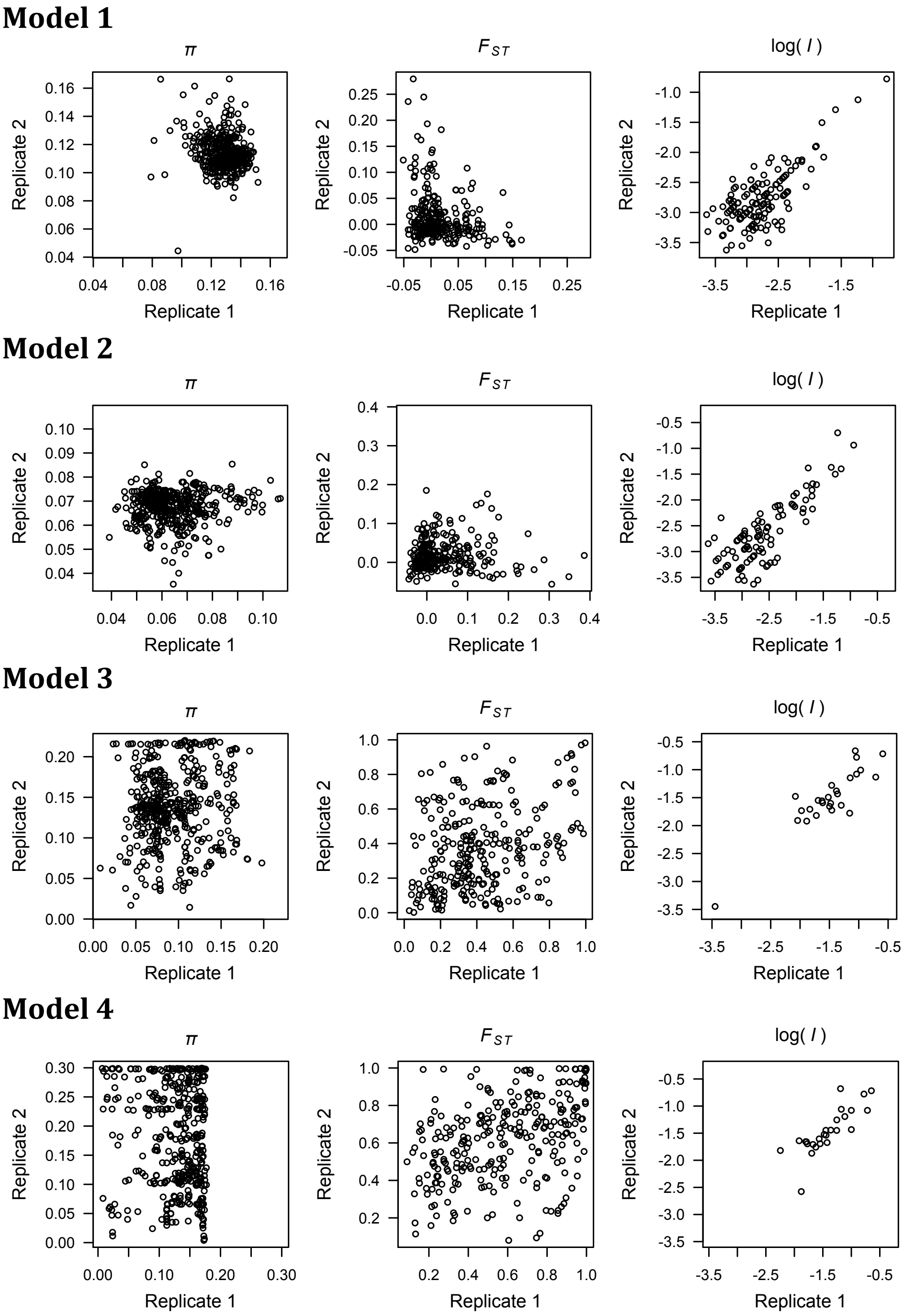

Figure S7. The stochastic variation of F_{ST} and π between simulation runs was higher than that of the proportion of nearly identical isolates (*I*) (1 SNP difference cutoff was used for nearly identical isolates here).

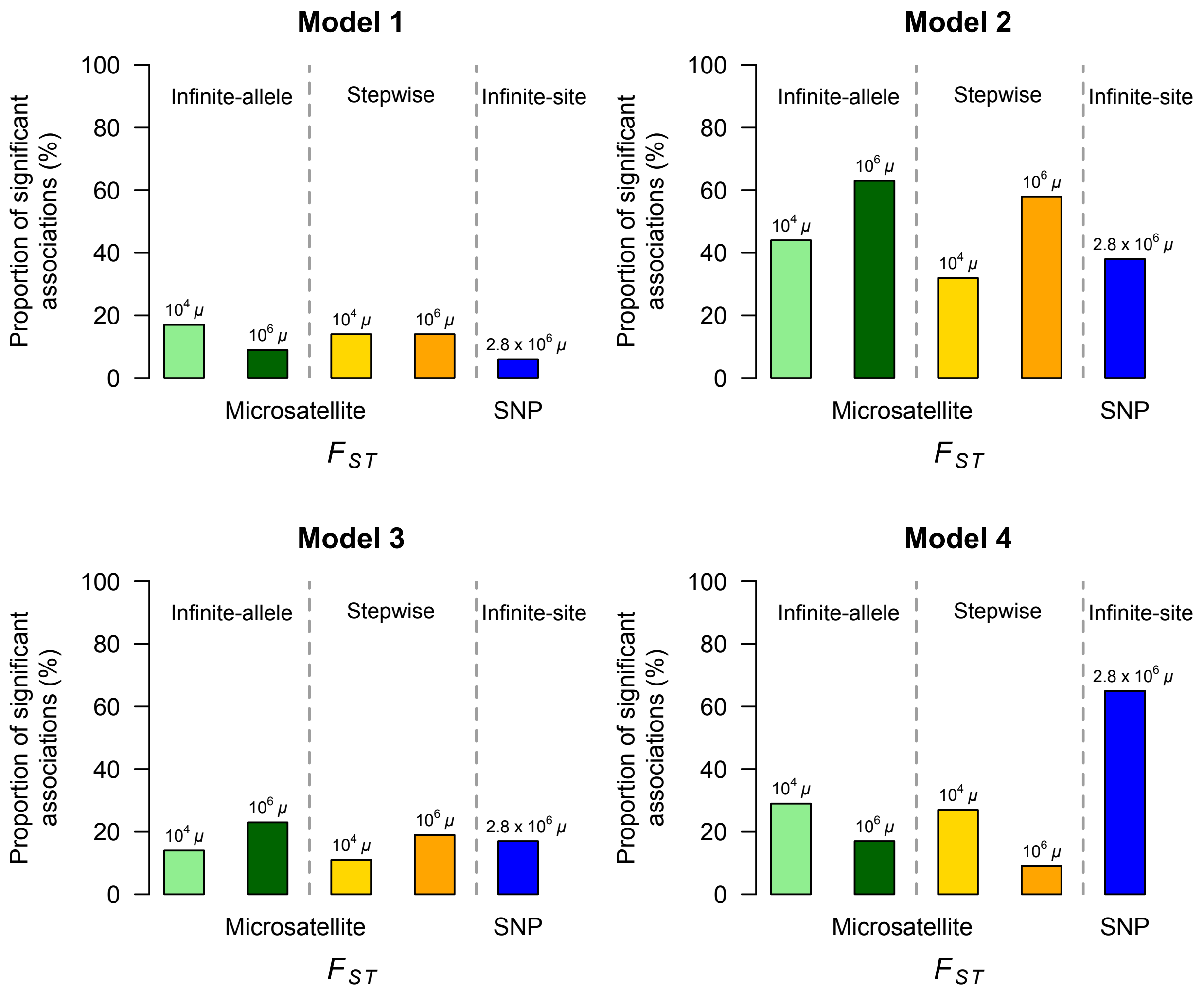

Figure S8. The power of F_{ST} calculated from microsatellite markers and genome-wide SNPs. All patient sharing was shifted by 0.00001 for the purpose of log-transformation. We used two different mutation rates ($10^4\mu$ and $10^6\mu$) in the microsatellite model. The per-site point mutation rate μ= 2.857 × 10^{-6} per year; figures above bars denote total mutation rate per site (microsatellite) or per genome (SNP).

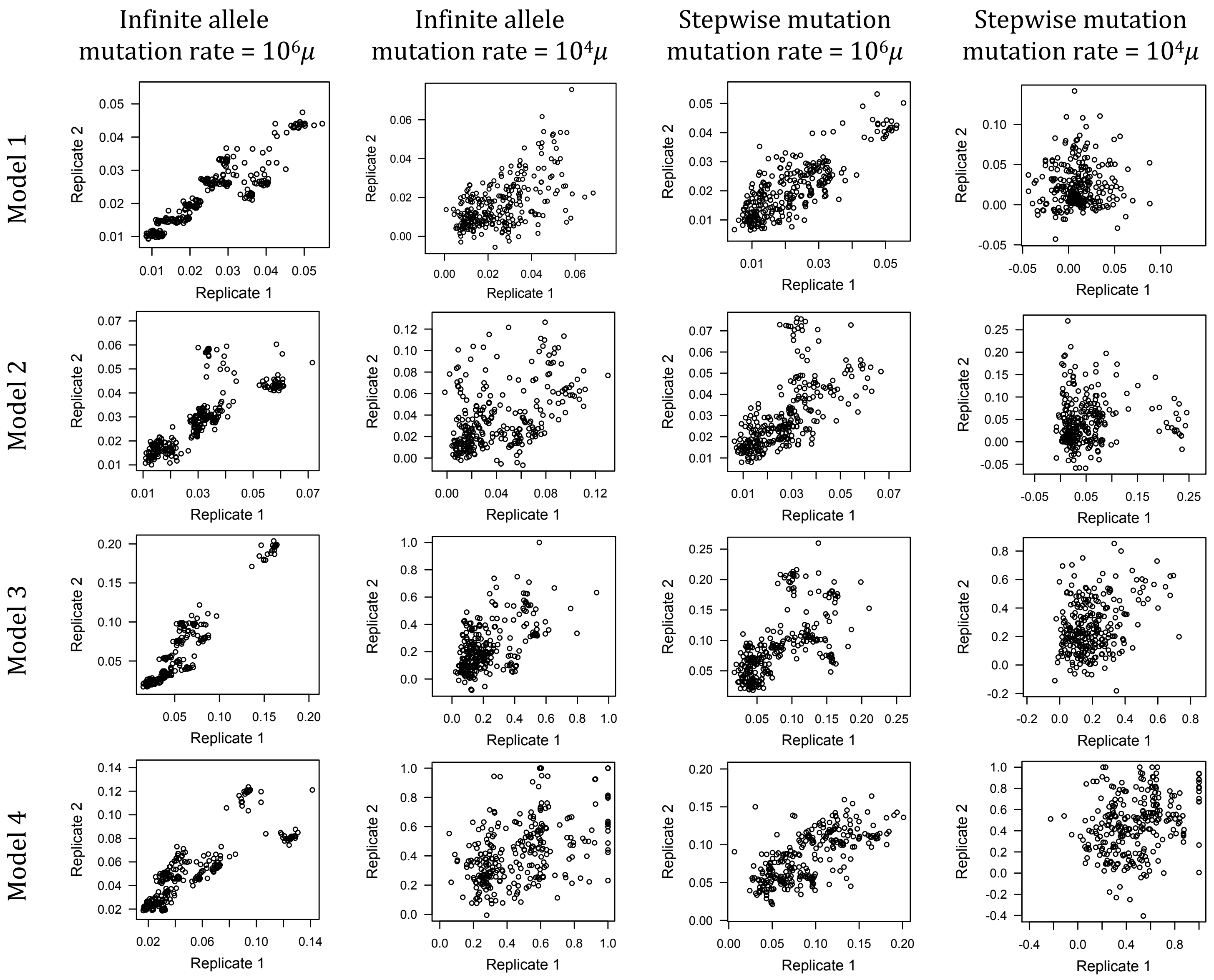

Figure S9. The stochastic variation of F_{ST} calculated from microsatellite markers was smaller than that calculated from SNPs (see Figure S6 for comparison).

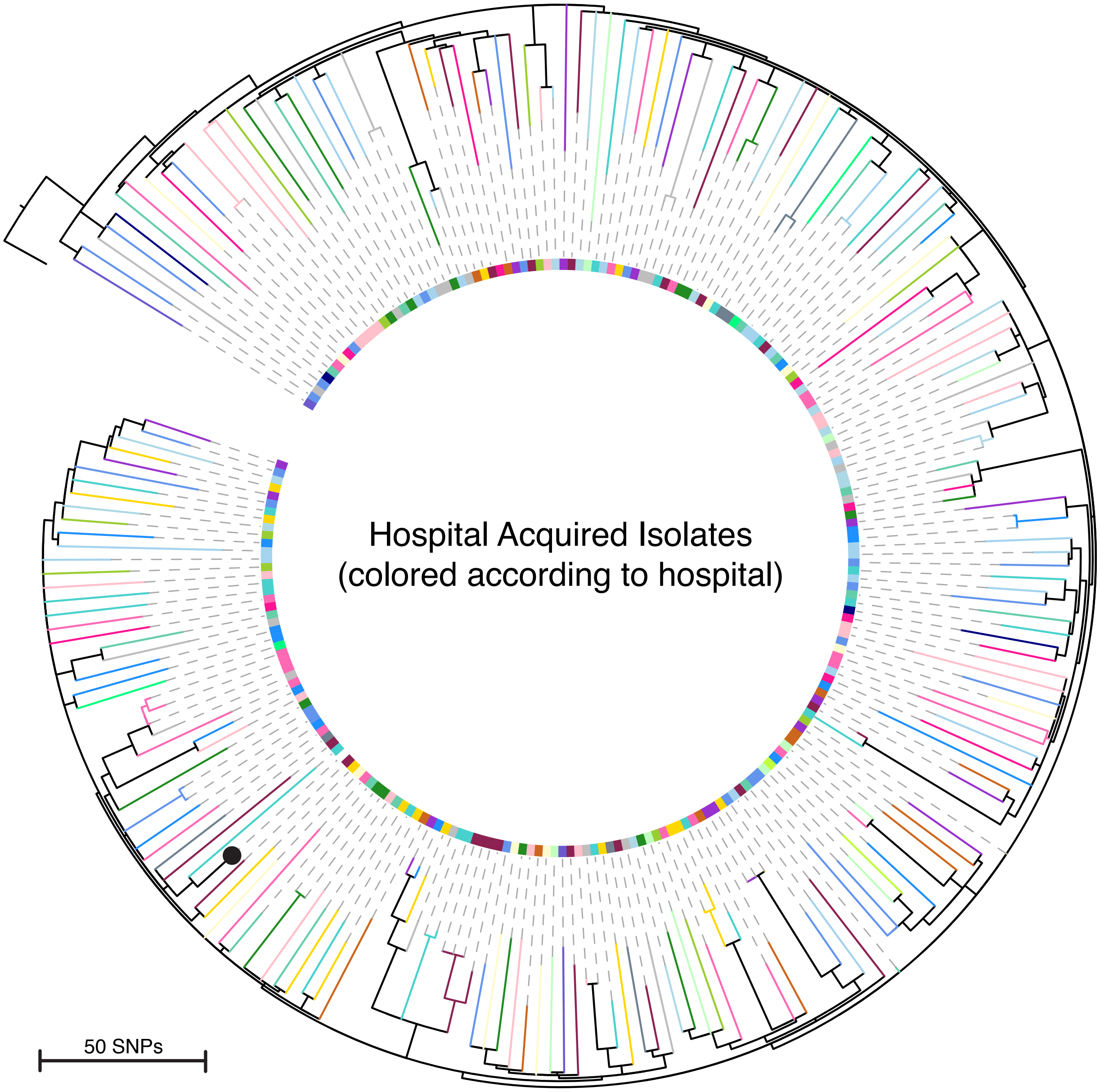

Figure S10. Phylogenetic tree after removing recombination.

Table S1. The sample size and proportion of community-onset isolates in each hospital

Hospital	Number of CO	Total sample size	Proportion of CO
1	12	16	0.75
2	45	60	0.75
3	18	20	0.90
4	47	64	0.73
5	11	13	0.85
6	57	68	0.84
7	23	23	1
8	49	65	0.75
9	15	25	0.60
10	14	16	0.86
11	1	1	1
12	16	28	0.57
13	2	2	1
14	4	4	1
15	9	9	1
16	37	54	0.69
17	29	36	0.81
18	39	50	0.78
19	35	50	0.7
20	20	26	0.77
21	15	16	0.94
22	8	8	1
23	25	33	0.76
24	39	55	0.71
25	9	16	0.56
26	4	4	1
27	41	56	0.73
28	26	37	0.70
29	58	65	0.89
30	55	66	0.83

Table S3. Analysis before and after removing recombination

	All SNPs		Removing recombination	
Within-hospital pairwise difference				
	87bp	0.353%	83bp	0.335%
Between-hospital pairwise difference				
	88bp	0.357%	85bp	0.340%
The correlation between indegree and within-hospital genetic diversity				
	r	*p*-value	*r*	*p*-value
	0.583	0.0018	0.636	7.78×10^{-5}
The correlation between the proportion of patients from other hospitals and within-hospital genetic diversity				
	r	*p*-value	*r*	*p*-value
	0.501	0.0092	0.594	0.0004
The association between $\log(I)$ and $\log(M)$				
	r	*p*-value	*r*	*p*-value
	0.185	0.038	0.175	0.044
The association between F_{ST} and $\log(M)$				
	r	*p*-value	*r*	*p*-value
	-0.112	0.11	-0.146	0.061
The association between π and $\log(M)$				
	r	*p*-value	*r*	*p*-value
	0.085	0.20	0.101	0.15

1. Croucher NJ, Page AJ, Connor TR, Delaney AJ, Keane JA, Bentley SD, Parkhill J, Harris SR: **Rapid phylogenetic analysis of large samples of recombinant bacterial whole genome sequences using Gubbins.** *Nucleic Acids Res* 2015, **43:**e15.
